# Evaluation of children’s cognitive load in processing and storage of their spatial working memory

**DOI:** 10.3389/fpsyg.2022.918048

**Published:** 2022-09-08

**Authors:** Hsiang-Chun Chen, Chien-Hui Kao, Tzu-Hua Wang, Yen-Ting Lai

**Affiliations:** ^1^Department of Early Childhood Education, National Tsing Hua University, Hsinchu City, Taiwan; ^2^Department of Education and Learning Technology, National Tsing Hua University, Hsinchu City, Taiwan; ^3^Department of Physical Medicine and Rehabilitation, National Taiwan University Hsin-Chu Hospital, Hsinchu, Taiwan

**Keywords:** cognitive load, complex span task, pupil dilation, spatial working memory, subjective evaluation

## Abstract

Working memory performance affects children’s learning. This study examined objective (task performance), subjective (self-report), and physiological (pupil dilation) cognitive load (CL) while children completed a spatial working memory complex span task. Frist, 80 Taiwanese 11-year-olds (40 boys) who participated in Experiment 1 confirmed the suitability of the materials. Then, 72 Taiwanese 11-year-olds (35 boys) were assigned to high and low complexity groups to participate in Experiment 2 to test the study hypothesis. Children had to recall at the end of a dual-task list and answer two questions regarding the difficulty and mental effort involved in processing and storage. Their pupil diameters were recorded using an eye-tracker. Two-way mixed ANOVA found that the processing requirements and memory load reduced storage and aggravated the subjective CL of storage; the subjective CL of processing was higher under highly complex conditions. Stepwise regression analysis indicated that subjective CL of processing predicted memory performance in low CL conditions, and physiological CL of processing predicted it in high CL conditions.

## Introduction

Working memory is a cognitive system that is responsible for information processing and temporary storage in daily life and learning (Miyake and Shah, 1999; Baddeley, 2003). Since working memory capacity is limited in children, it has been an important individual difference variable in exploring learning and cognitive processes. Research has shown that children’s working memory capacity is positively correlated with their academic achievement, reading comprehension, reasoning, and problem-solving abilities (Swanson and Jerman, 2007; Swanson et al., 2008; Swanson and Alloway, 2012). Therefore, many psychological and educational studies have been devoted to exploring the mechanisms of working memory, to understand the factors that lead to an increase in working memory capacity, and the impact they may have on the daily learning of children (Cowan, 1997; Gathercole, 2002).

Regarding the mechanism of children’s working memory, some studies contend that when storage and processing are performed simultaneously, they will compete for limited cognitive resources (Case et al., 1982). The time-based resource-sharing (TBRS) model proposed by Barrouillet et al. (2004) and Barrouillet et al. (2007) emphasizes that with the same processing time, the increasing difficulty in processing tasks will increase the cognitive load (CL) of the working memory system, thus resulting in poor memory performance. However, for a processing task that is objectively the same, the CL perceived by children may differ. Therefore, whether self-perceived CL generated by processing tasks affects children’s working memory performance remains to be clarified.

The data reflecting the psychological CL generated by individuals performing complex cognitive tasks have mainly been collected through written communications, observations, and interviews (Sweller et al., 1998). In the educational psychology literature, Sweller et al. (1998) divided the results drawn from CL into two dimensions. The task-based dimension measures the load that learners perceive while completing a task. It is called “mental load” and is related to the intrinsic (e.g., the difficulty of the material itself) and extrinsic characteristics (e.g., the presentation of the material) of the task. The learner-based dimension measures the cognitive energy exerted by learners while performing tasks. It is called “mental effort.” When the degree of difficulty or mental effort perceived by individuals is quantified numerically, the larger the numerical value, the greater the perceived CL (Paas and van Merriënboer, 1994).

In addition to self-reporting, physiological measurements can reflect the CL perceived by an individual. Many studies have treated pupil dilation as a “direct measurement of psychological activity” and have stated that it can distinguish between high and low CL caused by cognitive tasks (e.g., Hess and Polt, 1964; Unsworth and Robison, 2015). When the difficulty of a task increases, the overall pupil dilation also increases (Boersma et al., 1970). Participants with higher pupil dilation performed better than those with lower pupil dilation (Rondeel et al., 2015). The positive correlation between pupil dilation and behavioral performance indicates that the former reflects the additional effort required to improve the latter. Elucidating whether the pupil dilation of children performing processing tasks is related to their effort and working memory performance will provide insight into the mechanism of children’s working memory.

To our knowledge, a measure of CL in working memory complex span tasks from three different dimensions simultaneously, namely, the cognitive (behavioral performance), psychological (subjective perception), and physiological (pupil dilation) measurements, or the relationship among the three, has never been studied in children.

## Theory, measurement, and developmental changes of working memory

There are many theoretical models of working memory (Cowan, 1988; Engle et al., 1999; Miyake and Shah, 1999; Baddeley, 2000). The multi-component working memory model proposed by Baddeley (2000) has been widely applied in the study of developmental psychology and education (Swanson and Alloway, 2012). It emphasizes the importance of coordination among working memory subsystems (the central executive, visuospatial sketchpad, phonological loop, and episodic buffer) and the relationship between working and long-term memory (Baddeley, 2000). The central executive performs multiple functions, including allocating limited cognitive resources to other subsystems, maintaining, and switching attention, and suppressing the interference of irrelevant information (Baddeley et al., 2001). The visuospatial sketchpad and phonological loop are responsible for storing and processing visual–spatial and auditory-verbal information, respectively. The episodic buffer can temporarily store information from the visuospatial sketchpad and phonological loop and integrate it with activated information from long-term memory in real time (Baddeley, 2000).

Complex span tasks are among the predominantly used tools to measure working memory capacity. They are designed according to the construct of working memory, where participants must respond to processing tasks (e.g., verify whether the mathematical equation is true), remember the to-be-remembered (TBR) items and their sequences of storage tasks (e.g., the locations of dots in a grid), and recall the TBR items after several processing and storage tasks are alternately presented (Miyake et al., 2001). Complex span tasks increase difficulty gradually according to the number of TBR items (i.e., set size 2–5). The same span contains two or three trials. Participants continue the task if they recall any of the trials correctly within the same span. The maximum number of correctly recalled TBR items measured this way constitutes the participants’ working memory span (McNamara and Scott, 2001).

The developmental increase in working memory spans has often been attributed to an age-related increase in processing efficiency (Case et al., 1982). Towse and Hitch (1995) proposed a task-switching hypothesis, in which children switch their attention between processing and storage during working memory span tasks, and memory traces suffer from a time-related decay when children are engaged in the processing task. Therefore, higher working memory span in older children is attributed to their faster processing, resulting in shorter retention of the memory items. However, the TBRS model (Barrouillet et al., 2004; Barrouillet and Camos, 2015) stressed that processing and storage within complex span tasks rely on the same limited attention resource. Therefore, processing tasks that continuously occupy attention, thus preventing micro-task switching and the refreshment of memory traces, would involve a high CL and lead to poor working memory performance. Specifically, the CL that the processing task involves is the proportion of time during which this task occupies attention (Barrouillet et al., 2007). Thus, for the same length of time, the more times (or steps) of cognitive processing that must be completed in the processing tasks, the higher the CL, and the worse the children’s memory performance (Barrouillet and Camos, 2015). Older children would be more likely to exploit the pauses between processing tasks and switch their attention back and forth from processing to storage (Barrouillet et al., 2009, 2015).

## Classification and measurement of cognitive load

Sweller et al. (1998) put forward the CL theory from the perspectives of education and learning, asserting that CL is the load evoked when a specific task is applied to learners’ cognitive systems. CL theory explores how people can effectively deal with complex problems and acquire complex knowledge and skills through the coordinating ability of working and long-term memory. It divides CL into intrinsic, extraneous, and germane CL (Sweller et al., 1998). Whereas intrinsic CL is influenced by the difficulty of materials and learners’ experience, it is not related to the presentation of materials. Extraneous CL is influenced by the design or presentation of materials. Germane CL is influenced by all the extra information provided by an instructional designer or learning activities that meet individual needs. It appears to increase CL and can enhance cognitive efforts among learners so that they can construct and automate the schema. We expect children’s memory performance to be affected by the difficulty of processing tasks, that is, the inherent CL.

Wierwille and Eggemeier (1993) proposed methods to measure three types of CL, namely task-and performance-based, subjective, and physiological techniques. Task-and performance-based techniques infer the participants’ mental effort from objective task difficulty and behavioral performance results (e.g., scores and error rates). Generally, the higher the complexity of a task and worse the participants’ performance, the greater the task-evoked CL.

Subjective techniques measure the CL that participants perceive while reviewing and performing tasks. Generally, a rating scale is used to quantify mental effort into different grades. The participants assigned a grade score for perceived CL. The higher the value, the greater the perceived CL. The 9-point scale can reflect the self-perceived CL with little difference more sensitively (e.g., Paas et al., 1994; Gimino, 2002).

Physiological techniques record physiological changes that reflect the participants’ cognitive processes and take their physiological responses as indicators to measure CL, for example, through eye movements, heartbeats, breath, and brain waves. Eye movement research uses pupil dilation as an index of effort in cognitive tasks (van der Wel and van Steenbergen, 2018 for review). Pupil dilation reflects psychological activity. Therefore, the degree of pupil dilation increases with the difficulty of the task (Boersma et al., 1970; Klingner et al., 2011; Hogervorst et al., 2014). Unsworth and Robison (2015) recorded the dynamic process of changes in participants’ pupil size during working memory tasks using eye trackers and studied 70 college students’ performance on colored-square tasks. The pupil dilation value was calculated by subtracting the initial from the continuous pupil diameter of each participant during the task. The results showed that pupil dilation was affected by the number of TBR items and working memory capacity and reached the asymptote and approached stability after showing four TBR items.

## The present study

In this study, children’s CL and effort were measured with objective (task performance), subjective (self-report), and physiological (pupil diameter) tools when they performed complex span spatial working memory tasks. This study tested the hypothesis that besides the objective CL of processing tasks’ complexity, children’s self-perceived and physiological CL generated by processing tasks also affects their working memory performance. Specifically, the present study examined (1) the effects of processing task complexity and memory set sizes on children’s working memory performance, subjective perception, and pupil dilation; and (2) the impact of subjective and physiological CL on children’s working memory performance.

In our complex span task, the processing task was interleaved with a memory task. We used a between-subject design in which the children were randomly assigned into two groups (with high and low CL, respectively) to complete the processing task. Based on the TBSR model, we predicted that the children’s working memory performance under high CL processing tasks would be significantly worse than those under low CL processing tasks. Since self-perceived CL reflects task difficulty and mental effort, we predicted that children with higher self-reported CL levels would have worse working memory performance. In this study, while the children performed the complex span task, their eye movements and pupil diameters were recorded. If the degree of pupil dilation reflected additional effort to improve behavioral performance, then we predicted a significant positive correlation between pupil dilation and memory performance. This was only in the case of high processing complexity and more memory items; moreover, pupil dilation can predict memory performance.

**Figure 1:**
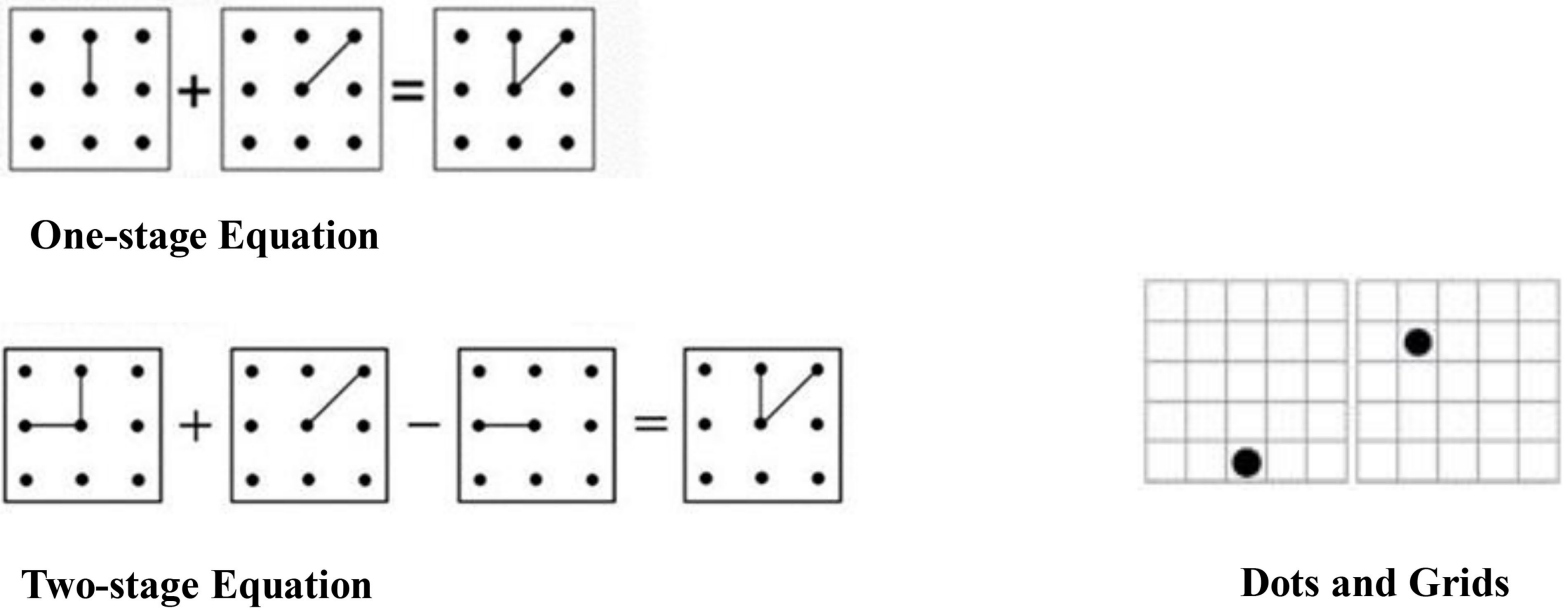
Illustration of stimulus in the spatial equation matrix and dot memory tasks.

The TBRS model focused on the working memory development of children aged 8–14 years (Lépine et al., 2005; Barrouillet et al., 2009). In addition, a CL review study (Mutlu-Bayraktar et al., 2019) indicated that 11–year–old children could complete the self-reported CL measurements. Therefore, this study selected 11–year–old children as the target in our two experiments. Considering the processing task of our experiments was a spatial matrix equation task initially designed for adults (Figure 1, adapted from Miyake et al., 2001, Dot Matrix), Experiment 1 was designed to examine whether the difficulty of the processing task used in this study was suitable for child participants. The mean reaction time of the child participants in Experiment 1 was used as the presentation time of the processing tasks in Experiment 2.

## Experiment 1

Experiment 1 was performed among 11-year-olds to test whether the difficulty of the processing tasks was suitable for child participants.

### Methods

#### Participants

Eighty 11-year-olds (40 boys) in one public elementary schools in urban areas in northern Taiwan were randomly assigned into two groups to participate in experiment 1. After instructions from researchers, children received tests in the classroom volunteering. Each child took about 5 min to complete the task and received a set of stationery as a reward for completing the task.

#### Materials and procedure

The spatial-matrix equation tasks (Figure. 1) was adapted from Miyake et al. (2001) Dot Matrix. Children completed the spatial-matrix equation tasks (Figure. 1) (one with two-stage equation, and the other with one-stage equation, each with 25 trials) in a pen-and-paper format (fill in T for “True” and F for “False” for the spatial-matrix equations). In the one-stage equation, the children judged whether a spatial-matrix pattern was correct or incorrect based on the addition or subtraction of a line segment. In the two-stage equation, children made the same judgment, but there was both addition and subtraction.

### Results

There was no significant difference between children’s accuracy in the two-stage equation task (*M* = 0.97, *SD* = 0.05) and that in the one-stage equation task (*M* = 0.97, *SD* = 0.04), *t*(78) = 0.314, *p* = 0.76, *Se* = 0.01, *d* = 0.07. However, the reaction time of the two-stage equation task (*M* = 6.67, *SD* = 1.64) was significantly longer than that of the one-stage equation task (*M* = 3.55, *SD* = 1.11), *t*(78) = 10.00, *p* < 0.001, *Se* = 0.31, *d* = 2.24.

These results indicated a difference in the complexity between the two tasks, and the difficulty was suitable for children aged 11 years. According to the average reaction time (*M* = 5.11, *SD* = 2.09) of the two tasks, the presentation time of processing tasks in our spatial-span self-evaluation tasks was set at 5 s.

## Experiment 2

Experiment 2 aimed to test the hypothesis that children’s self-perceived and physiological CL generated by processing tasks also affects their working memory performance besides the objective CL of processing task complexity. Experiment 2 had three predictions: (1) the children’s working memory performance under high CL processing tasks would be significantly worse than those under low CL processing tasks, (2) children with higher self-reported CL levels would have worse working memory performance, and (3) in the case of high processing complexity and more memory items, there is a significant positive correlation between pupil dilation and memory performance.

### Methods

#### Participants

A total of 72 11-year-olds from 2 public elementary schools in urban areas in northern Taiwan volunteered to participate in this study after their teachers verbally invited them. All children were randomly assigned into two groups (according to participation sequence and gender). Thus, 34 (16 boys, *M* = 11.58 years, *SD* = 0.28) and 38 (19 boys, *M* = 11.68 years, *SD* = 0.36) children participated in the high and low-complexity groups, respectively. The children received individual tests in a quiet and undisturbed meeting room during the school’s computer classes. Each child spent approximately 20 min on the task and received a stationery set as a reward when they completed it.

#### Materials

The spatial-span self-evaluation task was adapted from Miyake et al. (2001) Dot Matrix. It included processing (spatial-matrix equation task), memory (dot memory task), and self-evaluation tasks. There was one between-group manipulation of the complexity of the processing task (Figure. 1) (one-stage equation as low-complexity condition, two-stage equation as high-complexity condition). The processing task was interleaved with a memory task in which dots appeared in sequence inside a 5 × 5 square grid. The children had to remember this sequence and report it at the end of a list of two to three items. After each list, the children answered two questions pertaining to the difficulty of the processing task and the amount of effort invested in the memory task. The children’s eye movements and pupil diameters were recorded through the eye-tracker when they engaged in the spatial-span self-evaluation task.

#### Task and performance measurement

The children received three trials each of set sizes 2 and 3. Each trial of set size 2 included two each of processing and memory tasks. Each trial of set size 3 included three each of processing and memory tasks. Each memory task includes one dot on the grid. A total of 15 dots (2 × 3 + 3 × 3 = 15) appeared in sequence at a specific location in the grid, and each dot was fixed with a line-segment-equation. Whereas all children received the same stimulus sequence, the complexity of the processing tasks varied. The mean accuracy of the processing and recall tasks were calculated for each set size for further analysis.

#### Subjective measurement

The self-report tasks included “self-report of difficulty” (*How difficult was it for me to verify these equations?*) and “self-report of effort” (*How much effort did I spend remembering these black dots?*). Two self-report questions were presented after each trial. Each self-report question was rated on a 9-point scale, that ranged from 1 (*very simple or very little*) to 9 (*very difficult or very much*). The children answered by pressing numbers 1 to 9 on the computer keyboard. Each set size included three each of processing (self-report of difficulty) and memory task self-report questions (self-report of effort). We calculated the mean self-report scores of processing and memory tasks for each set size for further analysis.

#### Physiological measurement

GP3HD (GP3HD-16,493,388, produced by Gaze Point Research Inc., Canada) eye-tracker (sampling rate 150 Hz) was used to record the pupil size of the children when they performed spatial-span self-evaluation tasks. The children’s eye movements were monitored and recorded by a computer, and the stimuli were presented on the screen (21.3-inch, 1920 × 1,080) of another computer. The children were 65 cm away from the eye-tracker and screen, and the horizontal line of sight of both eyes was at 1/2 to 2/3 of the height of the screen (calculated from bottom to top). The stimulus in the processing task was divided into one- (4° × 1.9°) and two-stage (5.7° × 1.9°) equations. The stimulus in the memory task was a dot at a location in a 5 × 5 grid (13.3° × 13.3°). The average pupil diameter of the left and right eyes was measured when a child watched the “+” starting point in the first trial after the instruction. This was taken as the initial value of the child’s original pupil diameter. Pupil dilation (final pupil diameter minus the original pupil diameter) was calculated to analyze the pupil data (Unsworth and Robison, 2015). As the pupil diameter varied very minimally, the pupil dilation data were rounded off to the fourth decimal place for analysis. The mean pupil dilation of processing and recall tasks were calculated for each set size for further analysis.

#### Procedure

**Figure 2:**
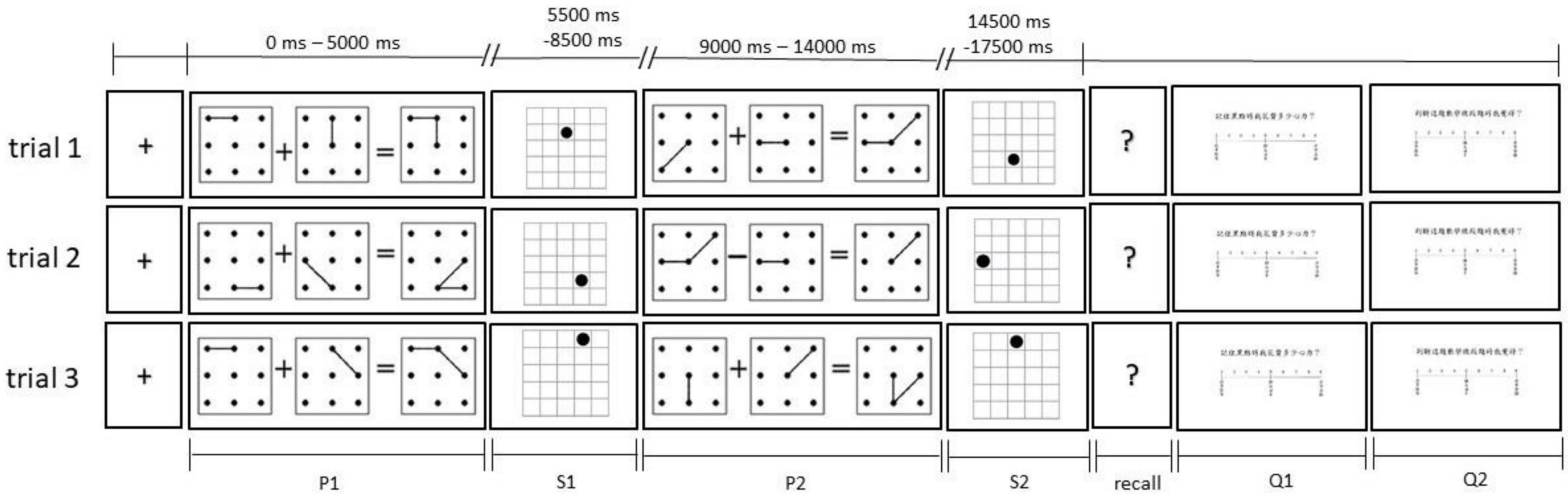
Experimental process (taking span 2 of the one-stage equation as an example). P, processing task. S, storage task; 1 or 2 refers to the position of the episodes in the trial. Q1, How much effort did I expend to remember these dots? Q2, How difficult was it for me to verify these equations?

Each child sat in a chair in front of the eye-tracker and performed a standard 9-point calibration (over 80% was acceptable). After the instructions and two practice trials, the experiment began. First, a “+” starting point appeared at the center of the computer screen. When the child was ready, the experimenter pressed the space key, and the matrix equation appeared on the screen for 5 s. The child was asked to verbally verify whether it was true or false within 5 s (the processing task). Then, a blank screen appeared for 500 milliseconds, followed by a grid with a dot for 3 s. The child was asked to remember the location of the dots and grids (storage task). When a question mark appeared on the screen, the child had to guess the location of the dots in the grid and mark their positions with a pen on a grid answer sheet. After each recall trial was completed, the screen presented two questions in sequence: “*How much effort did I expend to remember these dots*?” and “*How difficult was it for me to verify these equations?*’ The child was asked to press a number key to report the perceived CL grades. Following this, the next trial was conducted. Figure 2 illustrates the experimental process.

#### Design

A 2 × 2 mixed design with a between-subjects factor of processing task complexity (high and low) and a within-subjects factor for set size (2 and 3) were used. The dependent variables were the accuracy of the processing and memory tasks, self-report scores, and pupil dilation.

### Results

#### The descriptive statistics of the data

As denoted in Table 1, the skewness of all data is between-2.50 and 0.86, and the kurtosis is between-1.26 and 6.97, with skewness absolute value less than 3 and kurtosis absolute value less than 7, indicating that they do not deviate from the assumption of normal distribution (Byrne, 2010; Kline, 2015).

**Table 1:** Descriptive statistics of performance measurement, subjective measurement and physiological measurement.

Data source	Task	Set sizes	High-complexity condition (*N* = 34)				Low-complexity condition (*N* = 38)			
			Mean	SD	Skewness	Kurtosis	Mean	SD	Skewness	Kurtosis
Accuracy	Processing Task	2	0.77	0.21	−0.60	−0.54	0.95	0.11	−2.50	6.97
		3	0.78	0.16	−0.39	−0.66	0.95	0.08	−1.67	2.90
	Recall task	2	0.77	0.16	−0.11	−0.84	0.88	0.15	−0.91	−0.41
		3	0.68	0.22	−0.11	−1.26	0.79	0.16	−0.67	−0.20
Self-reported difficulty	Processing Task	2	4.21	1.57	0.29	0.03	2.40	1.48	0.84	−0.64
		3	4.49	1.85	−0.10	−0.90	2.33	1.41	0.78	−0.34
	Memory task	2	3.51	1.38	0.25	−0.80	2.78	1.52	0.52	−1.12
		3	4.34	1.75	−0.13	−0.69	3.52	1.56	0.40	−0.84
Pupil dilation	Processing Task	2	0.3463	0.3550	−1.59	5.07	0.3553	0.3087	0.32	0.68
		3	0.2999	0.3533	−1.33	5.00	0.3621	0.3699	0.64	1.25
	Memory task	2	0.3865	0.3575	−1.67	6.04	0.3824	0.3865	0.09	0.85
		3	0.3565	0.3789	−1.32	5.75	0.4237	0.4199	0.86	1.65

#### The effect of the cognitive load of the processing task complexity and the set sizes

Three methods were used to measure and analyze the effect of CL while performing a spatial-span working memory task.

#### Task and performance measurement

First, the mean accuracy of processing tasks was submitted to a 2 (processing task complexity: high and low) × 2 (set size: 2 and 3) two-way mixed measures ANOVA, which yielded a main effect of processing task complexity, *F*(1, 70) =37.15, *MSe* = 0.03, *p* < 0.001, *η_p_*^2^ = 0.35. The accuracy of the low-complexity condition (*M* = 0.95, *Se* = 0.02) was significantly higher than that of the high-complexity condition (*M* = 0.77, *Se* = 0.02). We did not find a main effect of set size. The processing task complexity × set size interaction was also not significant. The accuracy of the processing tasks is shown in Table 2.

**Table 2:** Mean (SD) of the subjective measurement and performance measurement in processing tasks.

Set sizes	Trials	High-complexity condition		Low-complexity condition	
		Self-reported difficulty	Processing task accuracy	Self-reported difficulty	Processing task accuracy
2	1	4.56 (1.86)	0.72 (0.41)	2.66 (1.89)	0.96 (0.14)
	2	4.00 (1.86)	0.75 (0.33)	2.32 (1.60)	0.92 (0.22)
	3	4.06 (1.74)	0.84 (0.27)	2.24 (1.40)	0.96 (0.14)
	Mean	4.21 (1.57)	0.77 (0.21)	2.40 (1.48)	0.95 (0.11)
3	1	4.35 (2.03)	0.76 (0.26)	2.56 (1.81)	0.93 (0.16)
	2	4.59 (1.78)	0.83 (0.25)	2.21 (1.47)	0.96 (0.11)
	3	4.53 (2.16)	0.75 (0.25)	2.24 (1.53)	0.96 (0.14)
	Mean	4.49 (1.85)	0.78 (0.16)	2.33 (1.41)	0.95 (0.08)

Then, the mean accuracy of the recall tasks was submitted to a two-way ANOVA, which yielded a main effect of set size, *F*(1, 70) = 16.69, *MSe* = 0.02, *p* < 0.001, *η_p_*^2^ = 0.19. The accuracy of set size 2 (*M* = 0.83, *Se* = 0.02) was significantly higher than that of set size 3 (*M* = 0.73, *Se* = 0.02). We also found a main effect of processing task complexity, *F*(1, 70) =10.65, *MSe* = 0.04, *p* = 0.002, *η_p_*^2^ = 0.13. The accuracy of the low-complexity condition (*M* = 0.83, *Se* = 0.02) was significantly higher than that of the high-complexity one (*M* = 0.73, *Se* = 0.02). There was no significant processing task complexity or set size interaction. The accuracy of the recall tasks is shown in Table 3. These results show that children’s performance in processing tasks was not affected by the CL of the set sizes, but only by that of the complexity of the processing tasks. Conversely, children’s performances in recall tasks were affected by the CL of the set sizes and of the complexity of the processing tasks.

**Table 3:** Mean (SD) of the subjective measurement and performance measurement in memory tasks.

Set sizes	Trials	High-complexity condition		Low-complexity condition	
		Self-reported effort	Recall task accuracy	Self-reported effort	Recall task accuracy
2	1	3.56 (1.74)	0.74 (0.28)	2.79 (1.70)	0.87 (0.24)
	2	3.62 (1.60)	0.76 (0.28)	2.84 (1.72)	0.87 (0.25)
	3	3.35 (1.59)	0.82 (0.30)	2.71 (1.56)	0.91 (0.23)
	Mean	3.51 (1.38)	0.77 (0.16)	2.78 (1.52)	0.88 (0.15)
3	1	4.68 (2.03)	0.73 (0.28)	3.87 (1.73)	0.77 (0.21)
	2	4.41 (2.02)	0.51 (0.34)	3.61 (2.07)	0.76 (0.26)
	3	3.94 (1.84)	0.78 (0.31)	3.08 (1.81)	0.82 (0.31)
	Mean	4.34 (1.75)	0.68 (0.22)	3.52 (1.56)	0.79 (0.16)

#### Subjective measurement

First, the mean self-reported difficulty of the processing tasks was submitted to a 2 (processing task complexity: high and low) × 2 (set size: 2 and 3) two-way mixed measures ANOVA, which yielded a main effect of processing task complexity, *F*(1, 70) = 30.92, *MSe* = 4.55, *p* < 0.001, *η_p_*^2^ = 0.31. The self-reported difficulty of the high-complexity condition (*M* = 4.35, *Se* = 0.26) was significantly higher than that of the low-complexity one (*M* = 2.37, *Se* = 0.02). We did not find a main effect of set size. The processing task complexity × set size interaction was also not significant. The self-reported difficulty of processing tasks is shown in Table 2.

Then, the mean self-reported effort of the memory tasks was subjected to a two-factor ANOVA, which yielded a main effect of processing task complexity, *F*(1, 70) = 4.94, *MSe* = 4.39, *p* = 0.03, *η_p_*^2^ = 0.07. The self-reported effort in the high-complexity condition (*M* = 3.93, *Se* = 0.25) was significantly higher than that in the low-complexity one (*M* = 3.15, *Se* = 0.24). We also found a main effect of set size, *F*(1, 70) = 48.83, *MSe* = 0.45, *p* < 0.001, *η_p_*^2^ = 0.41. The self-reported effort of set size 3 (*M* = 3.93, *Se* = 0.20) was significantly higher than that of set size 2 (*M* = 3.15, *Se* = 0.17). The processing task complexity × set size interaction was not significant. The self-reported effort of memory tasks is shown in Table 3.

These results show that children’s perceived CL of processing tasks was not affected by the set sizes, but by the complexity of the processing tasks. Conversely, children’s perceived CL of memory tasks was affected by the set sizes and complexity of the processing tasks.

#### Physiological measurement

First, the mean pupil dilation of the processing tasks was submitted to a 2 (processing task complexity: high and low) × 2 (set size: 2 and 3) two-way mixed measures ANOVA. No main effects or interactions were found. The pupil dilation of the processing tasks is shown in Table 4.

**Table 4:** Mean (SD) of pupil dilation during processing and memory tasks.

Set sizes	Trials	High-complexity condition		Low-complexity condition	
		Processing tasks	Memory tasks	Processing tasks	Memory tasks
2	1	0.4010 (0.3636)	0.3799 (0.4053)	0.3863 (0.3553)	0.3519 (0.3267)
	2	0.3469 (0.3741)	0.4326 (0.3665)	0.3223 (0.4177)	0.3918 (0.4613)
	3	0.2910 (0.3674)	0.3470 (0.3622)	0.3572 (0.3634)	0.4249 (0.4275)
	Mean	0.3463 (0.3550)	0.3865 (0.3575)	0.3553 (0.3087)	0.3824 (0.3865)
3	1	0.3143 (0.3858)	0.4093 (0.3623)	0.3727 (0.3690)	0.4757 (0.4483)
	2	0.2885 (0.3332)	0.3541 (0.3929)	0.3611 (0.3857)	0.4030 (0.4393)
	3	0.2969 (0.3655)	0.3061 (0.4077)	0.3526 (0.3881)	0.3923 (0.4141)
	Mean	0.2999 (0.3533)	0.3565 (0.3789)	0.3621 (0.3699)	0.4237 (0.4199)

The mean pupil dilation of the memory tasks was subjected to a two-way ANOVA. No main effects were observed. The processing task complexity × set size interaction was significant, *F*(1, 70) = 5.04, *MSe* = 0.01, *p* = 0.03, *η_p_*^2^ = 0.07. However, the post-comparison analysis did not reveal any other significant differences. The pupil dilation of the memory tasks is shown in Table 4.

#### Summary

Performance and subjective measurement showed that processing tasks with greater complexity and memory tasks with larger set sizes led to higher CL. The accuracy and self-reported difficulty of processing tasks were affected by their complexity. The accuracy and self-reported effort of the memory task were not only affected by the set sizes or the complexity of the processing task. However, physiological measurements using pupil dilation did not reveal clear CL effects.

#### The impact of subjective and physiological cognitive load on task performance

##### Correlation analysis

First, Pearson’s correlation analysis was conducted to examine the correlation between self-reported CL and task accuracy. In the low-complexity condition at set size 2, the children’s self-reported difficulty and effort were significantly negatively correlated with the accuracy of the memory tasks (*r* = −0.51, *p* = 0.001; *r* = −0.45, *p* = 0.005). Their self-reported difficulty was significantly negatively correlated with the accuracy of the processing tasks (*r* = −0.33, *p* = 0.045). No other significant correlations were found. These results indicate that when the complexity of processing tasks is low and the set size is small, children with greater subjective CL have poorer task performance.

Pearson’s correlation analysis was conducted to examine the correlation between pupil dilation and task accuracy. In the high-complexity condition at set size 3, the children’s pupil dilation in processing and recall tasks were significantly positively correlated with the accuracy of the recall tasks (*r* = 0.41, *p* = 0.02; *r* = 0.36, *p* = 0.03). However, in the low-complexity condition of set size 2, the children’s pupil dilation in processing tasks was significantly negatively correlated with the accuracy of the processing tasks (*r* = −0.40, *p* = 0.01). These results showed that when the complexity of processing tasks was high and the set size was large, children with greater pupil dilation had better memory performance. In contrast, when the complexity of the processing task was low and the set size was small, the greater the pupil dilation, the worse the performance in the processing task.

##### Stepwise regression analysis

Stepwise regression analysis was carried out to examine whether subjective or physiological CLs were significant predictors of working memory task performance. Two analyses were conducted with the accuracy of the processing and memory tasks as dependent variables, and self-reported difficulty and effort, and pupil dilation in the processing and memory tasks as predictor variables. The outcomes of the stepwise regression analyses are summarized in Table 5. Pupil dilation in the processing task as a physiological CL measure contributed to the prediction of the processing task performance in the low-complexity condition at set size 2, adjusted *R*^2^ = 0.14, *F* (1, 37) = 6.87, *p* = 0.01, and the memory task performance in the high-complexity condition at set size 3, adjusted *R*^2^ = 0.14, *F*(1, 33) = 6.57, *p* = 0.02. Self-reported difficulty as a subjective CL measure contributed to the prediction of the memory task performance in the low-complexity condition of set size 2, adjusted *R*^2^ = 0.29, *F*(1, 37) = 12.72, *p* = 0.001.

**Table 5:** Results of the stepwise regression analysis of CL measures.

Dependent variables	Predictor variables	*B*	*SE*	*β*	*t*
*Processing task performance*					
Low-complexity condition, set size 2	Pupil dilation of processing task	−0.14	0.06	−0.40	−2.62
*Memory task performance*					
High-complexity condition, set size 3	Pupil dilation of processing task	0.25	0.10	0.41	2.56
Low-complexity condition, set size 2	Self-reported difficulty	−0.05	0.02	−0.51	−3.57

In some conditions, the subjective and physiological CL of processing task complexity was correlated with task performance and emerged as significant predictors. However, subjective and physiological CL of the memory task had no significant predictive effect on task performance.

## Discussion

This study examined objective (task performance), subjective (self-report), and physiological (pupil dilation) measures of CL, while children completed a complex span visuospatial working memory task. The results indicate several patterns. We review and discuss our findings based on (1) measuring the CL of processing and storage in a complex span task, and (2) the impact of subjective and physiological CL on working memory performance.

### Measuring the cognitive load of processing and storage in the complex span task

#### The task and performance measurement

First, the accuracy of the processing task was significantly lower in the high-complexity condition than in the low-complexity one, indicating an objective performance decrement at a higher CL. Second, accuracy in the memory task was significantly lower in the high-complexity condition than in the low-complexity one and significantly lower in set size 3 than in set size 2. These results indicated that processing requirements and memory load reduced working memory storage.

According to the TBRS theory, Barrouillet et al. (2004, 2007) thought that with the same time limit, the more complicated the processing task, the higher the CL and the worse the children’s memory performance. In this study, the processing tasks were fixed for five seconds. Our results are in accordance with previous working memory studies conducted on adults (Barrouillet et al., 2004, 2015) and children (Barrouillet et al., 2009; Oftinger and Camos, 2018).

#### Subjective and physiological measurement

In terms of the subjective and physiological measures of CL, first, compared to the low-complexity condition, children self-reported greater difficulty in the processing task in high-complexity conditions at all set size levels. Second, children in the high-complexity condition and at the high set size level reported significantly higher effort to remember the dot locations than in the low-complexity condition and at the small set size level. This indicates that more processing requirements and memory items aggravated the subjective CL of storage in working memory. Third, pupil dilation did not present a clear pattern.

Research on CL assessment showed that the greater the value reported by learners, the greater the perceived CL (Paas and van Merriënboer, 1994). According to the CL theory (Sweller et al., 2011), intrinsic CL is experienced owing to the complexity of the learning task itself. Our results suggest that children consciously perceive the CL caused by processing and storage in their working memory. Such measures are sufficiently sensitive to the discrimination of the CL of working memory span tasks.

Processing tasks with high complexity made children aware of the higher CL of memory tasks (greater effort). This result echoes the task performance, reflecting the competition of attention resources for processing and storage in children’s working memory (Cowan, 2010; Vergauwe et al., 2010; Barrouillet et al., 2015). When processing consumes more attention resources, there are fewer attention resources left for storage, which leads children to consciously perceive that they need more mental effort to complete their memory tasks.

However, this was not the case with pupil dilation. Our results showed that children’s pupil dilation was not affected by processing task complexity or memory set size. Adult eye movement studies have found that pupil dilation increases when the CL of a task increases (Klingner et al., 2011). For example, in the Stroop task, incongruent stimuli induced larger pupil dilation than did congruent stimuli (Laeng et al., 2011; Rondeel et al., 2015). Heitz et al. (2008) found that when adults kept items in their working memory, the degree of pupil dilation was proportionate to the number of items they needed to remember. Pupil dilation should be larger at larger set sizes, indicative of the CL imposed by remembering more information (Unsworth and Robison, 2015). We did not find significant changes in the children’s pupil dilation with CL conditions, which may have been the result of between-subject manipulation of processing task complexity and the small set sizes in the memory tasks.

### The impact of subjective and physiological CL on working memory performance

Research on working memory has focused on the influence of the CL of processing tasks on children’s performance in memory tasks (Barrouillet et al., 2009; Oftinger and Camos, 2018). Our study explored the impact of subjective and physiological CL on the processing and storage of working memory. Correlation and stepwise regression analysis of working memory performance under different conditions revealed that children’s subjective perception of CL and pupil dilation evoked by the processing task predicted their working memory performance.

#### Subjectively perceived CL and task performance

In the low-complexity condition of set size 2, the children’s subjective CL of processing was negatively correlated with and predicted their memory performance. The children’s subjective CL of processing and memory were negatively correlated with memory performance, but only subjective CL of processing predicted their memory performance. To the best of our knowledge, this study is the first to examine the effect of children’s subjective CL on working memory performance. Our findings showed that children’s subjective perceptions of task difficulty and mental effort were related to actual performance. Children’s subjective perceptions of task difficulty negatively predicted working memory performance when the CL on processing and storage was not heavy.

#### Physiological CL and task performance

First, in the low-complexity processing task at set size 2, the children’s pupil dilation during processing was negatively correlated with and predicted their processing performance. Second, in the high-complexity processing task at set size 3, children’s pupil dilation during processing and storage was positively correlated with their memory performance. However, only pupil dilation during processing predicted memory performance in children. These indicate that enough attention and experience of CL would lead to better performance of a high complexity task and lead to the worse performance of a low complexity task.

These findings are consistent with those of recent research on adults (Rondeel et al., 2015; Robison and Unsworth, 2019). It was confirmed that participants with larger pupil dilation performed better on cognitive tasks than those with smaller pupil dilation (Rondeel et al., 2015). Robison and Unsworth (2019) found that when participants performed visual–spatial working memory tasks, the trials with better performance (remembering four memory items) had larger pupil dilation than those with poorer performance (remembering two memory items). Other studies found that when the participants reported that they were in an off-task attentional state, the task-evoked pupil dilations were small, and their performance was poor (e.g., the reaction time was longer) (Unsworth and Robison, 2016, 2018; Unsworth et al., 2018). This study compared pupil dilation evoked by different CL conditions of processing and storage in a complex span task and their predicted effect on task performance, examined in children.

### Limitations and future research

We used immediate ratings of CL and asked children to report perceived difficulty and mental effort by pressing numeric keys immediately after each list in the complex span task (e.g., Paas and van Merriënboer, 1994; Dindar et al., 2015), instead of using delayed ratings of CL, which requires children to recall and report the CL caused by the task after completing all working memory span tasks (e.g., Leutner et al., 2009; Inan et al., 2015). The main reason for this is that the immediate evaluation method can accurately determine the CL perceived by children caused by ongoing work. In contrast, the delayed evaluation method cannot confirm whether the estimation of the CL reported by children comes from the average, the last work, or the most complex work (Anmarkrud et al., 2019). However, this study could not prevent children from being distracted by monitoring the perceived CL during processing or storage tasks, which interfered with the operation of working memory (Anmarkrud et al., 2019).

The task performance and the subjective and physiological CL were compared between children at processing tasks with different complexities to avoid the practice effect caused by repeated tests and interference with children’s self-perception of CL. A within-subject design can be adopted in future research to control for individual differences more strictly among participants. This study is the first to systematically analyze the CL of working memory reflected by behavioral, psychological, and physiological dimensions. Future research can consider the spatial-span self-evaluation task designed by our study to measure children’s CL in working memory.

## Data availability statement

The raw data supporting the conclusions of this article will be made available by the authors, without undue reservation.

## Ethics statement

Ethical review and approval was not always required for studies on human participants in accordance with the local legislation and institutional requirements. Written informed consent from the participants’ legal guardian/next of kin was not required to participate in this study. This study complies with the declaration of Helsinki concerning the recruitment of participants, data collection, data use, data confidentiality, and protection of participants’ rights.

## Author contributions

H-CC and T-HW contributed to conception and design of the study, and organized the database. H-CC and C‐HK performed the statistical analysis. H-CC, T-HW, and C‐HK wrote the first draft of the manuscript. Y-TL contributed to the data analysis and interpretation of the results in the manuscript revision process based on the reviewer comments. All authors contributed to manuscript revision, read, and approved the submitted version.

## Funding

The authors deeply appreciate the Ministry of Science and Technology in Taiwan for the financial support and encouragement under grant no. 108-2410-H-007-048, 105-2511-S-007-014-MY3, and 106-2511-S-007-003-MY3.

## Conflict of interest

The authors declare that the research was conducted in the absence of any commercial or financial relationships that could be construed as a potential conflict of interest.

## Publisher’s note

All claims expressed in this article are solely those of the authors and do not necessarily represent those of their affiliated organizations, or those of the publisher, the editors and the reviewers. Any product that may be evaluated in this article, or claim that may be made by its manufacturer, is not guaranteed or endorsed by the publisher.
